# Funicular Myelosis in a Butcher: It Was the Cream Cans

**DOI:** 10.1155/2015/827168

**Published:** 2015-01-28

**Authors:** Fabian Wolpert, Krisztina Baráth, Janis Brakowski, Roland Renzel, Michael Linnebank, Andreas R. Gantenbein

**Affiliations:** ^1^Department of Neurology, University Hospital Zürich, Frauenklinikstrasse 26, 8091 Zürich, Switzerland; ^2^Medizinisch Radiologisches Institut (MRI Bethanien), Toblerstrasse 51, Bahnhofplatz, Stadelhofen, 8044 Zürich, Switzerland; ^3^Department of Psychiatry, Psychotherapy and Psychosomatics, Hospital of Psychiatry, University of Zurich, Lenggstrasse 31, 8032 Zürich, Switzerland; ^4^Neurorehabilitation, RehaClinic, Quellenstrasse 34, 5330 Bad Zurzach, Switzerland

## Abstract

*Background*. Funicular myelosis is a known consequence of exposure to nitrous oxide. Nevertheless, there are only a few clinical trials assessing its long-term effects and there is no literature about the role of nutritional vitamin B12 supplementation in the context of nitrous oxide abuse. *Case Descriptions*. We diagnosed funicular myelosis in a young butcher, who consumed high amounts of meat regularly. Since the diagnostic process did not reveal any metabolic causes, reinterrogation of the patient uncovered recreational abuse of nitrous oxide out of whipped cream can gas cartridges. After stopping abuse and supplementation of vitamin B12, the patient recovered almost completely. *Conclusions*. In our case, even high nutritional vitamin B12 uptake could not compensate the noxious effects of nitrous oxide. Since there are emerging reports of increasing misuse, this should be considered in the diagnostic and therapeutic care of patients with nitrous oxide abuse. Furthermore, our case emphasizes that patients with vitamin B12 deficiency should be assessed for nitrous oxide abuse.

## 1. Case Presentation

The 27-year-old patient, a butcher, presented with a 4-week history of ascending symmetric numbness in the limbs, as well as tingling in the feet and fingers. He felt clumsy while writing and unsecure during walking, with report of several dropping events. The neurological examination revealed a diminished position and vibration sense and hypalgesia and hypaesthesia of the upper and lower limbs, compatible with distal symmetric polyneuropathy. However, the Romberg test was negative and the remainder of the examination was normal; in particular, there were no weaknesses or neuropsychological symptoms.

**Figure 1:**
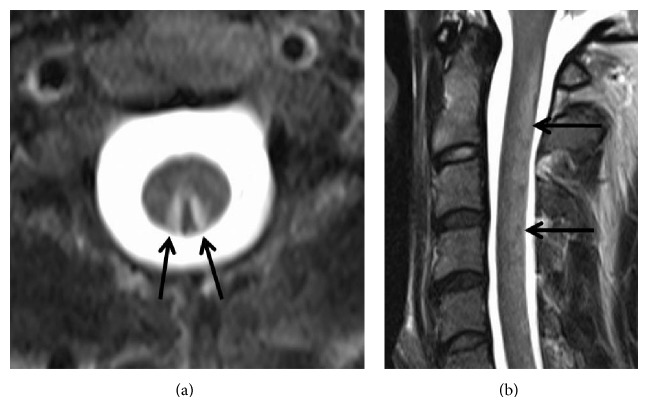
The T2-weighted axial (a) and sagittal (b) images of the cervical spine show hyperintensity (arrows) of the dorsal columns of the spinal cord. There was no contrast enhancement of the lesion (not shown).

The MRI of the brain was normal. The spinal MRI showed T2-hyperintense lesions in the dorsal columns of the cervical spine (C1–C6) without contrast enhancement as typical in patients with funicular myelosis [1] (Figure 1). Elevated homocysteine plasma levels (^*^106.0 *μ*mol/L [5–13.5]) in addition to decreased serum vitamin B12 levels (136 ng/[180–900]) confirmed the diagnosis of funicular myelosis due to vitamin B12 deficiency (Table 1). Other blood tests were normal; in particular, there was no megaloblastic anaemia, as often seen in patients with alimentary deficiency of vitamin B12 [2]. The patient was consuming meat at least once to twice a day. Therefore, an alimentary cause of vitamin B12 deficiency was unlikely. The patient reported to be eupeptic and without gastric symptoms. Duodenogastroscopy including duodenal and gastric biopsies did not reveal signs of atrophic gastritis or other possible causes for impaired vitamin B12 resorption. Serology was negative for anti-intrinsic factor or anti-parietal cell antibodies. Reenquiry of the patient and his mother revealed a recreational abuse of nitrous oxide (N_2_O) in the previous months. He acquired N_2_O from gas cartridges used in whipped cream cans.

The patient was treated with intramuscular vitamin B12 (hydroxocobalamine, 1000 *μ*g for 6 days and thereafter 100 *μ*g per week) and oral folic acid. He was prescribed regular physical and occupational training and was advised to stop abusing nitrous oxide. At follow-up, after 4 weeks, there was still a slightly disturbed joint-position sense in the right toe. Bimalleolar vibration sense had improved from 1/8 to 5/8 as assessed by the scale of the vibration tune (0 no sense, 8 full vibration sense). Otherwise, neurological examination was normal. He went back to work.

## 2. Discussion

Vitamin B12 deficiency, subsequent hyperhomocysteinaemia, and funicular myelosis have been observed in patients after exposure to nitrous oxide, and the underlying biochemical pathomechanism has been revealed. It indicates an irreversible inhibition of the active cobalt centre of vitamin B12, leading to decreased activity of 5-methyltetrahydrofolate-homocysteine methyltransferase (MTR), a vitamin B12 dependent enzyme converting homocysteine to methionine [3].

In addition to several case reports, a small study revealed that long-term nitrous oxide exposure in operating theatres may lead to decreased vitamin B12 serum levels [4]. Interestingly, similar to the findings in our case, no significant haematological changes such as megaloblastic anaemia were observed in those patients.

Besides this abuse of nitrous oxide and the resulting degenerative effects (myelopathy and peripheral neuropathy), Cousaert et al. [5] stated an increasing misuse of nitrous oxide. The overall abuse prevalence in adolescents and young adults was reported to vary between 12% and 20% in different studies [5]. In their overview, they focused on occurring psychiatric symptoms and described cases of mild mood disorders, psychotic behaviour, fatigue, generalized weakness, and loss of memory. These symptoms often preceded the neurological impairments and were most probably caused by vitamin B12 deficiency as they decreased after high dose treatment with vitamin B12. Nevertheless, little is known about the exact neurobiological development mechanisms, the severity of the symptoms over time, or the degree of abuse. Therefore, venturous therapy suggestions like using nitrous oxide as a treatment for depression instead of electroconvulsive therapy (ECT) because of its beneficial effect (inducement of laughter, central sympathetic stimulation, and release of endogenous opioid peptides) [6] should be viewed with caution. Furthermore, because of its euphoric, anxiolytic, and narcotic effects, nitrous oxide has a high potential of dependency. Single cases of death by addictive inhalation of this substance have been described [7], and, in a large study of narcotic drug abuse deaths by the Office of the Chief Medical Examiner of Maryland between 1991 and 2006, single use of volatile drugs like nitrous oxide caused 9,4% of 149 deaths [8]. These findings show the importance of a critical controversy about the continued misuse of nitrous oxide and its consequences.

We consider this report to be worth publishing, as we found a unique constellation, where even high nutritional vitamin B12 uptake with a diet rich in meat was not able to compensate the noxious effects of N_2_O. To our knowledge, there are no controlled studies available that concern the influence of nutritional supply of vitamin B12; therefore, the present case suggests that alimentary increased vitamin B12 uptake may not prevent onset of funicular myelosis upon nitrous oxide exposure. This may be of clinical importance when assessing risks for patients with abuse of or other reasons of long-term exposure to nitrous oxide. Furthermore, the case emphasizes that patients (and their next kin) should be asked for nitrous oxide exposure or abuse in cases of vitamin B12 deficiency of unknown origin.

## Figures and Tables

**Table 1:** The laboratory results showing typical changes indicating vitamin B12 deficiency.

	Unit	Value	Normal range
Homocysteine	*μ*mol/L	106.0^*^	5–13.5
Folic acid	*µ*g/L	5.6	2.5–9.0
Vitamin B12	ng/L	136^*^	180–900
Holotranscobalamin	pmol/L	18^*^	>37
Anti-parietal cell antibodies	U/mL	0	<10
Anti-intrinsic factor antibodies	U/mL	0.5	<6.0
Mean corpuscular volume (MCV)	fL	88.4	80–100
Hemoglobin	g/dL	15.6	13.4–17
